# An Adolescent With a Migrated V‐Shaped Wire Into the Neck: A Case Report

**DOI:** 10.1002/ccr3.71582

**Published:** 2025-12-02

**Authors:** Zi Ye, Xuechun Zhai, Xin Bian, Pingli Yang

**Affiliations:** ^1^ Department of Otolaryngology The First Affiliated Hospital of Shihezi University Shihezi China

**Keywords:** adolescent, case presentation, foreign body, removal, suspension laryngoscope

## Abstract

This case highlights the importance of high‐resolution imaging and intraoperative navigation for diagnosing and managing rare cases of migrated foreign bodies in the retropharyngeal space. It also underscores the criticality of timely foreign body removal and presents a viable approach for extraction under suspension laryngoscope, offering valuable reference for managing similar future cases.

## Graphical abstract

This abstract figure delineates the diagnostic and therapeutic workflow for a patient with a rare case of migrated foreign bodies in the retropharyngeal space, the treatment strategy we opted for, as well as the patient's prognosis.

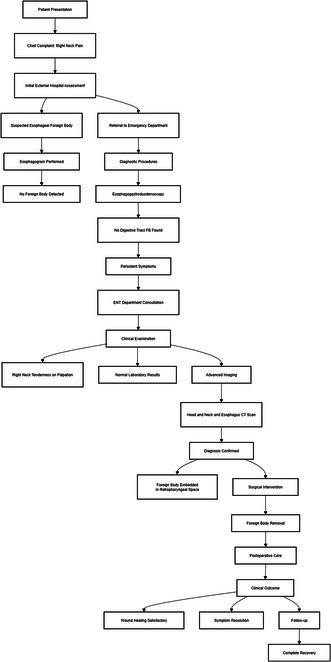

## 1 Introduction

Foreign body ingestion is a common clinical occurrence worldwide, with high morbidity in the pediatric population and in adult patients [1]. According to the American Association of Poison Control Centers' annual report for 2011, over 95,000 foreign body ingestions were reported in children, with > 3000 of those in patients 13–19 years of age [2]. Clinical manifestation varies depending on the impaction sites, the types and sizes of the ingested foreign bodies, the time elapsed following ingestion, and complications [3]. In adults, foreign body upper aerodigestive tract is a common presentation, but foreign body migrating into the retropharyngeal space is not only uncommon, but its management also differs and is challenging too, which is usually caused accidentally [4]. Here, we have described an unusual case of a foreign body (a V‐shaped wire) in the retropharyngeal space of an adolescent patient.

## 2 Case History/Examination

A 15 year‐old male adolescent with no known comorbidities presented to the Emergency Department of The First Affiliated Hospital of Shihezi University (Shihezi, China) on September 30, 2024, due to foreign body sensation in the throat and odynophagia after eating noodles for 1 day. The patient complained of experiencing pain during swallowing, pain in the right side of the neck, and a significant worsening of the pain when moving his head. On physical examination, he was conscious, oriented, and cooperative, hemodynamically stable without any signs of respiratory distress. His axillary temperature was 37.2°C. There was no obvious redness, swelling, or subcutaneous emphysema on the surface of the patient's neck skin. The right lateral neck exhibited palpable tenderness, and inspection showed minimal congestion of the throat mucosa.

The patient initially sought help at a nearby hospital when the patient first experienced discomfort the day prior, where an esophageal foreign body was suspected. An esophageal contrast study was performed at the external facility, but no foreign body was identified. The patient presented to the Emergency Department of our hospital the following day. The emergency physician at that time still considered the diagnosis of an esophageal foreign body. Since the patient had not yet undergone esophagogastroduodenoscopy (EGD)—a procedure capable of providing direct visualization from the pharyngeal entrance to the gastroduodenum; thus, the emergency physician recommended completing this examination in our Gastroenterology Department. During the EGD procedure, the patient experienced significant pain when the esophagoscope contacted his right tonsil, but no obvious foreign body was visualized under the endoscopic field. The patient's blood test results did not demonstrate any clinical or laboratory indicators suggestive of infection.

Gastroenterology Department investigations revealed no foreign body presence throughout the entire examination process. Given the persistence of severe subjective odynophagia and neck pain, the patient presented to the ENT (Ear, Nose, and Throat) outpatient clinic. Firstly, an electronic laryngoscope was arranged for him, and the image showed a small area of suspected protuberance that was noted on the right piriform fossa (Figure 2). The CT scan demonstrates higher sensitivity and specificity compared to anteroposterior/lateral chest X‐rays and provides critical anatomical localization should surgical intervention become necessary. Given the patient's fasting status and to minimize procedural discomfort, a noncontrast head–neck–esophagus CT scan was prioritized over conventional anteroposterior/lateral chest–abdominal radiographs, which revealed (Figure 2), a V‐shaped foreign body in the retropharyngeal space at the level of C4–5 that was obliquely oriented with the right end slightly superior, and the foreign body was above the thyroid and very close to the right carotid artery.

Although no bystanders witnessed the ingestion of a foreign body, the patient presented with severe clinical symptoms after swallowing food (notably odynophagia and exacerbation of symptoms with head rotation). Prior to consultation in our department, the possibility of an esophageal foreign body had been ruled out. However, during gastroscopy, the patient experienced significant pain upon palpation of the right pharyngeal tonsil, leading us to provisionally consider the persistence of a foreign body. The diagnosis of an ingested foreign body embedded in the neck was subsequently confirmed following a CT scan.

## 3 Differential Diagnosis

Based on the patient's medical history and relevant laboratory and imaging studies, we have ruled out the possibility of both traumatic foreign body and iatrogenic foreign body in the patient. Since the patient did not exhibit symptoms of dyspnea or a choking cough, we ruled out the possibility of a tracheal foreign body.

As bone impaction is the most common foreign body ingestion type, it is essential to distinguish between bone impaction and wire impaction. But in this case, the CT shows a V‐shaped high‐density shadow without any other structural features such as trabeculae of bone and the sensitivity of intraoperative lateral radiographs is nearly 100%; thus, we have excluded bone as the potential foreign object.

## 4 Outcome and Follow‐Up

**FIGURE 1:**
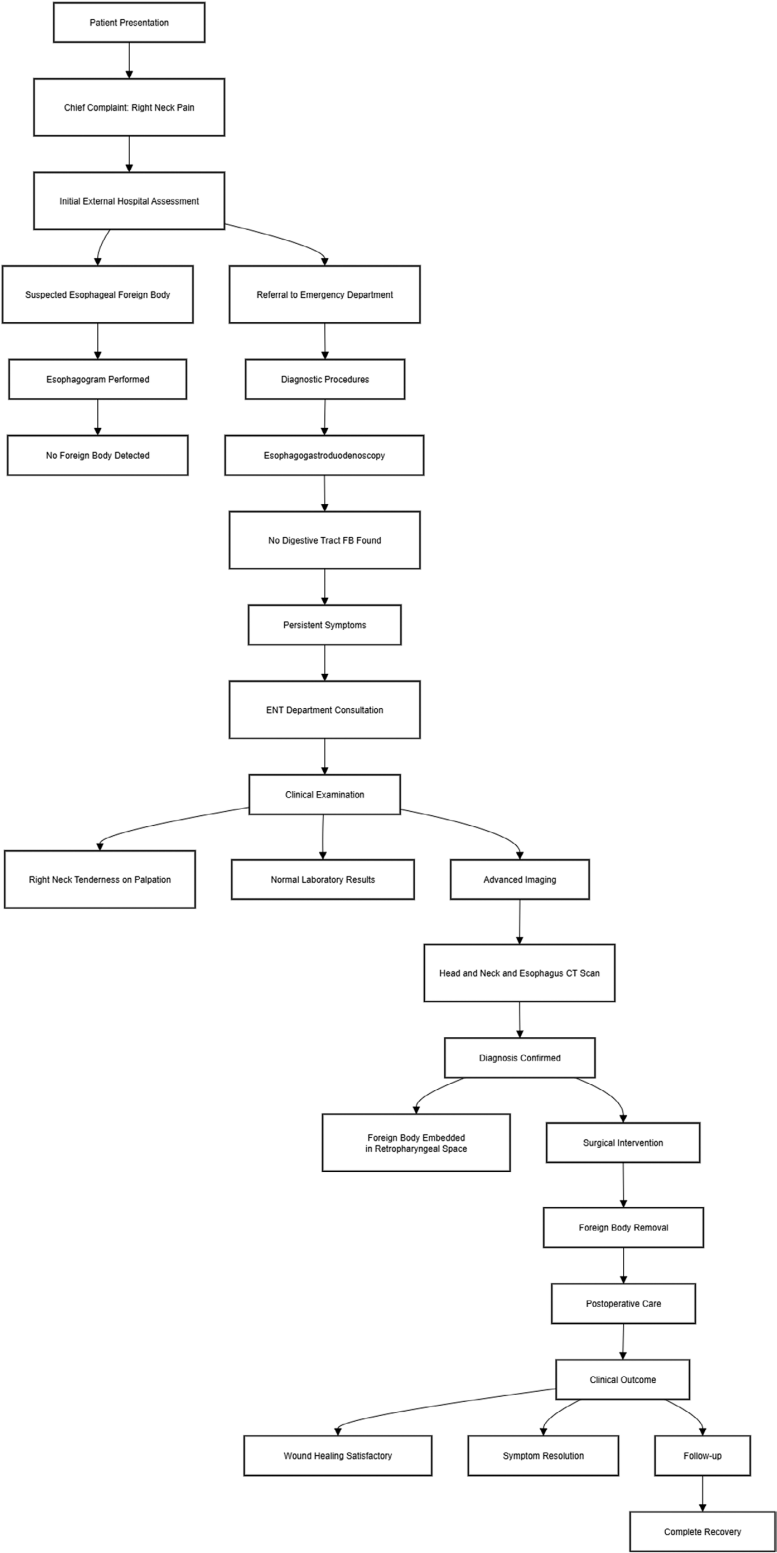
Flowchart of diagnostic and therapeutic process for the present case.

Based on the aforementioned diagnostic and treatment rationale and relevant investigations, we proceeded with surgical intervention. The flowchart in (Figure 1), delineates the entire diagnostic and therapeutic pathway.

**FIGURE 2:**
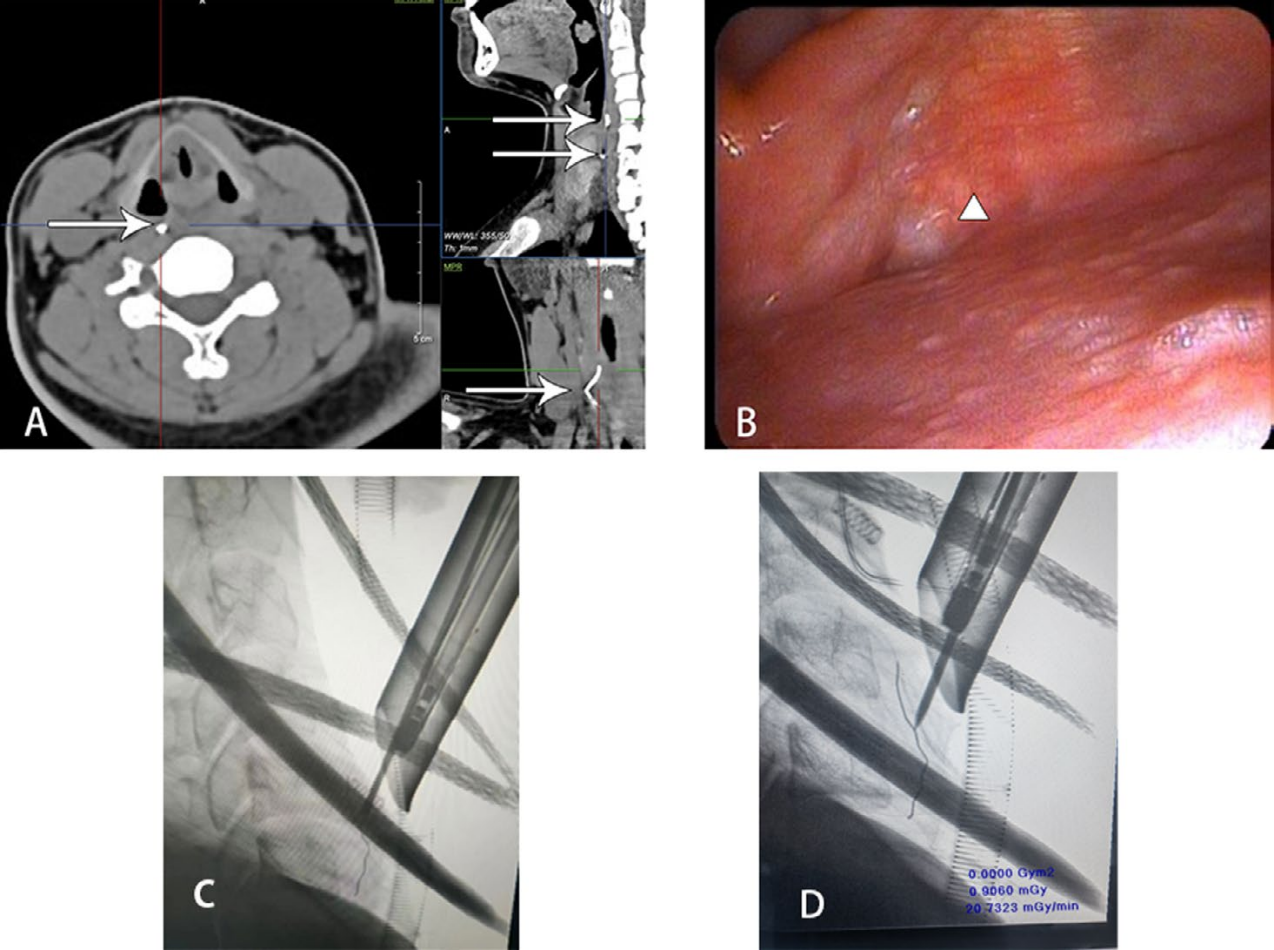
Imaging examination of the patient. (A) Neck CT confirmed the presence of an obliquely oriented density (arrow) in the retropharyngeal space at the C4–5 level. (B) The electronic laryngoscope image showed a small area of suspected protuberance was noted on the right piriform fossa (triangle). (C) The first lateral neck intraoperative X‐ray. (D) The second lateral neck intraoperative X‐ray.

**FIGURE 3:**
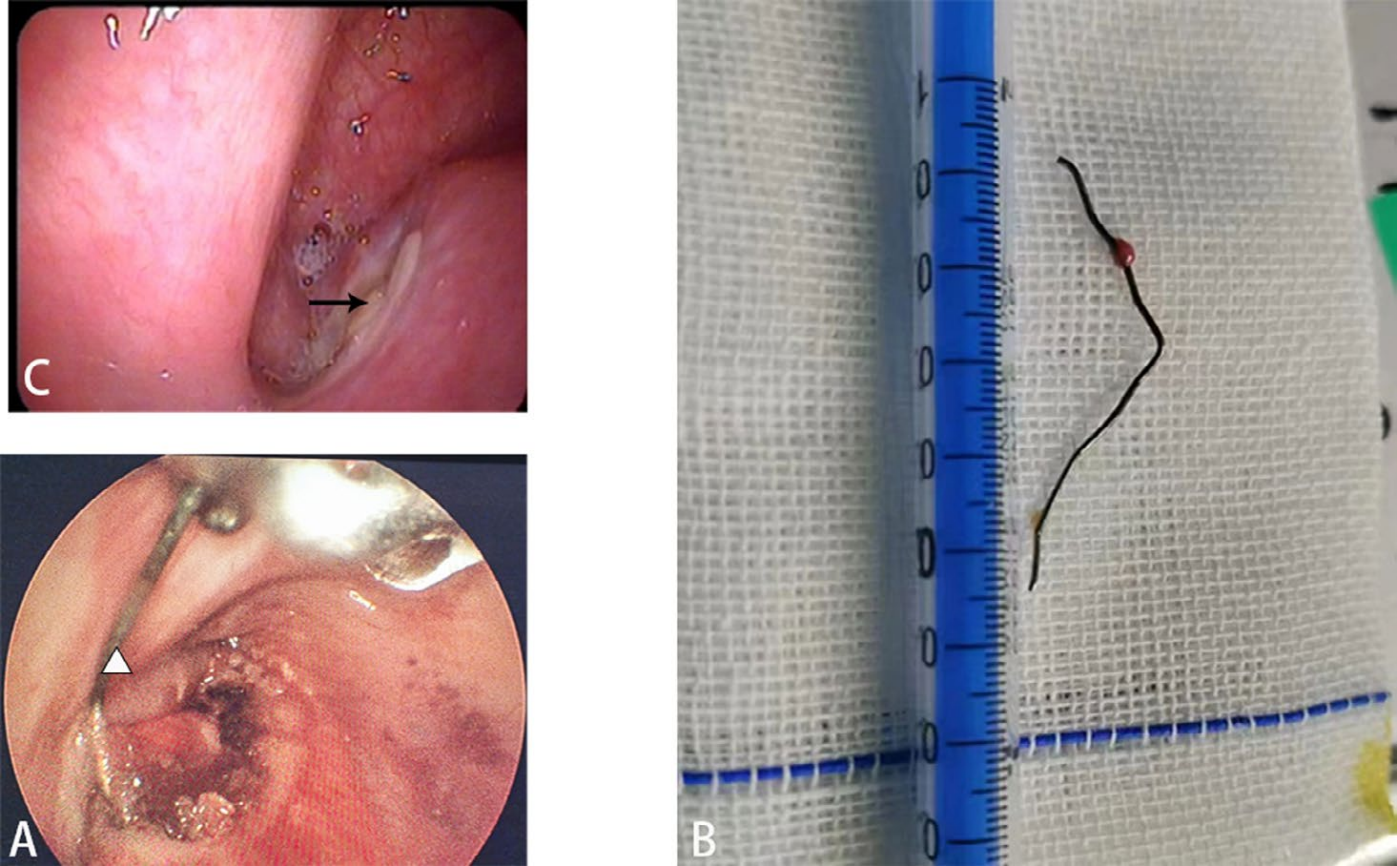
Removal of foreign body and follow‐up laryngoscopy imaging. (A) Successful removal of the foreign body was accomplished using laryngeal foreign body retrieval forceps (triangle). (B) Removed V‐shaped wire. (C) Laryngoscopy imaging on postoperative day 8 (POD 8) demonstrated satisfactory recovery, with the area of interest marked by an arrow.

After obtaining informed consent, we proceeded with the surgical procedure under general anesthesia with endotracheal intubation. The patient was placed in the supine position with cervical extension. Using a suspension laryngoscope, we performed a transoral examination and identified a suspicious elevated lesion in the right piriform sinus. While no foreign body was visualized intraoperatively, this region corresponded to the radiographic location of the suspected foreign body on preoperative CT imaging. Based on this correlation, we selected the right piriform sinus as the surgical access site. However, we were unable to obtain a clear anteroposterior view to confirm proper vertical alignment, which prevented us from making an incision to locate the foreign body in the tissue. Therefore, we made an intraoperative X‐ray image to affirm the accurate position of the foreign body. A thin V‐shaped black line was visualized which confirmed that the monopolar front‐end was touching near the foreign body (Figure 2). Under direct endoscopic visualization via a transoral rigid laryngoscope and following topical application of an adrenaline‐soaked pledget for hemostasis, a monopolar electrocautery device was employed to meticulously dissect the submucosal plane with precise thermal control. But the tip of the foreign body still could not be exposed clearly. Thus, a second intraoperative X‐ray image (Figure 2) was made. Intraoperative imaging confirmed contact between the monopolar electrocautery probe tip and the distal aspect of the foreign body. Under direct endoscopic visualization via a transoral rigid laryngoscope, a laryngeal foreign body retrieval forceps was introduced to securely grasp the exposed tip of the embedded foreign body, enabling controlled extraction from the right piriform sinus mucosa (Figure 3). The V‐shaped metal wire was removed completely (Figure 3), as confirmed on endoscope. The patient was extubated and sent to recovery in stable condition. Postoperative laboratory findings after the surgery indicated a mild infection in the patient (manifested by slightly elevated white blood cell and neutrophil counts).

Sufficient amounts of Cefuroxime Sodium (1 g, q8h, ivgtt normal) according to the patient's weight were administered intraoperatively and postoperatively for antibacterial treatment. On the 8th day after the operation, the patient returned to the hospital for an electronic laryngoscopy, which showed good recovery at the surgical site (Figure 3). The patient reported no pain during swallowing, no significant pain at the surgical site, and a reduction in neck pain compared to before. Considering the patient's reluctance to undergo repeated radiation exposure, he did not undergo neck and esophageal CT scans. Telephone follow‐up half a month after the operation showed satisfactory treatment effects without severe complications.

## 5 Discussion

Foreign body impaction in the throat is a very common condition and a common presenting complaint at the emergency and otolaryngology department [5]. The foreign body was more likely to enter the surrounding tissue from the tonsil area, the epiglottis valley, or the piriform fossa, and in this case, the foreign body can be speculated to move to the retropharyngeal space through the piriform fossa. A study [6], showed that at least one end of the foreign body was sharp, and all of them were slender. This shape is favorable for foreign bodies to penetrate the tissues and stay in the pharynx. If a patient tries to alleviate the symptoms by swallowing, it would stab into the tissue deeper until it completely falls into the submucous membrane and even migrates. When a foreign body enters its surrounding tissue, the damage to local tissue and the pathogenic bacteria it carries would cause an inflammatory reaction, and the local tissue would undergo a series of pathological changes such as metamorphosis, exudation, hyperplasia, and inflammatory mediators releasing [7, 8]. Therefore, the removal of foreign bodies was the key to treatment; otherwise, the persistent inflammatory response would cause the symptoms to persist and even cause retropharyngeal abscess. There have been case reports showing that it may occur potentially serious complications of retropharyngeal abscess, such as involvement of the jugular veins and carotid arteries or spread of infection resulting in deep neck space infection and/or mediastinitis [9]. Xu et al. [10], reported three cases where fish bones were swallowed and penetrated the esophagus, leading to deep neck infections. Open neck surgery was performed to remove the foreign bodies (fish bones) from the neck, thereby enabling incision and drainage of the abscesses. One case involved a patient who, 13 days after accidentally ingesting a fish bone and 6 days into antibiotic treatment, still developed retrosternal pain and a high fever of 44°C. Another case involved a patient who accidentally ingested a fish bone 9 days prior and, despite experiencing dysphagia, did not seek medical attention, subsequently developing symptoms such as fever, chills, and fatigue. A CT scan showed that the fish bone was protruding into the superior mediastinum and had formed a mediastinal abscess and left subclavian artery aneurysmal hematoma. This case report describes a V‐shaped foreign body located superior to the thyroid gland and in close proximity to the right common carotid artery. Without prompt surgical intervention, the potential for catastrophic complications—including vascular injury or airway compromise—remains a critical concern.

Most foreign bodies in the throat can be seen and removed easily. However, foreign bodies that migrated into the retropharyngeal space are not easily discovered. They may pose the potential for disastrous consequences [11]. X‐ray is limited by the two‐dimensional nature of projectional imaging, which precludes precise anatomical localization [12]. CT may be concerned about its radiation exposure, but the high‐density foreign body is in close proximity to the internal carotid artery, posing a significant risk of severe complications during any routine daily activities. Given the urgency of addressing this risk factor, we prioritized a CT scan with higher sensitivity and 3D reconstruction capabilities, thereby bypassing the standard X‐ray examination procedure.

A meta‐analysis found the overall sensitivity of lateral neck X‐rays to be 58% and the specificity to be 94% for detecting foreign bodies. However, lateral neck X‐rays' high specificity indicates a false negative would be unlikely. This has led physicians to search elsewhere for highly sensitive diagnostic imaging as the first line. CT scans were found to be highly sensitive, ranging from 85.7% to 100% and specificity, ranging from 66.7% to 100% [13].

CT can also be the best guiding tool for evaluating the treatment efficacy if complications like abscesses happen, and after neck and chest surgery, a CT scan has also become an important indicator to judge the recovery of the patient [14].

The reasons we did not attempt endoscopic resection and open exploration through an external cervical approach for diagnostic and therapeutic purposes are as follows: (1) The foreign body was embedded in the patient's neck, with no visible foreign body stump identifiable endoscopically. (2) Endoscopic incisional exploration was technically challenging under laryngoscopy in our hospital. (3) External cervical open exploration carries relatively greater morbidity risks, including postoperative infection/hematoma, prolonged recovery time, and significant patient discomfort. (4) CT imaging revealed a foreign body protruding from the cervical esophagus into the retropharyngeal space, positioned adjacent to the cervical esophagus. The transoral approach via suspension laryngoscopy offers minimal invasiveness to the patient, requiring less tissue dissection compared to the external cervical approach, resulting in reduced trauma and faster postoperative recovery. (5) Following a discussion of the associated risks with the patient's family, they opted for the less invasive approach of foreign body removal under suspension laryngoscopy.

The endoscopy is useful not only to visualize the collapsed mucosa but also to detect and expose the imbedded foreign body by sweeping the mucosa with the use of the suspension laryngoscope, and the suspension laryngoscope also makes sufficient space to operate the forceps to remove the wire in the collapsed space [15]. Further use of the suspension laryngoscope in patients having a history of foreign body ingestion in adolescents can help determine if this technology can be efficiently used in removing foreign bodies in the throat.

At present, several guidelines and systematic reviews have discussed this topic. Sahn et al. [16], mentioned that the management of adolescents with sharp object ingestion can be challenging for multiple reasons, including that the ingestion is intentional and/or repeated, and there are many controversies regarding how aggressive endoscopic or surgical removal of the object should be [17, 18]. There is a better consensus that nonstraight sharps, such as open safety pins and dual‐ended sharp objects, such as toothpicks or food bones, should be urgently removed if possible, with close inpatient observation if not retrieved [16]. In this case report, after a comprehensive assessment, we decided to remove the foreign body of the 15 year‐old adolescent by surgery rather than take close observation to avoid further complications.

In terms of the prevention of foreign body ingestion management in adolescents, Sahn et al. [16], mentioned in a review that repeated intentional ingestions and co‐morbid psychiatric illness are common in adolescents having a history of foreign body ingestion, and knowing the type of foreign body, the necessity of surgical removal, and potential complications may provide caregivers with heightened awareness for this occurring again in selected individuals.

A V‐shaped wire is rarely seen in the retropharyngeal space. But this case provides a complete process of removing such a challenging foreign body from the neck. This case demonstrates the following aspects: (1) Most of the time, the electronic laryngoscope is a visual way of the Department of Otolaryngology to check out if there exists any foreign body within the field of vision; however, if any sharp foreign body moves into the patients' neck, it will be challenging to find out using the electronic laryngoscope. (2) The upper gastrointestinal endoscopy is necessary to determine whether the foreign body has entered the digestive tract. (3) Emergent Computer tomography could be an accurate and effective way to confirm if there exists any foreign body, and it can also provide a relatively rough positioning. (4) It may be difficult to separate the tissues to search for the target; thus, the intraoperative X‐ray image could be helpful to better locate the target. (5) Suspension laryngoscopy is still a good way to remove a foreign body, especially those challenging cases.

## Author Contributions

**Zi Ye:** conceptualization, investigation, project administration, writing – original draft, writing – review and editing. **Xuechun Zhai:** supervision, validation. **Xin Bian:** resources, supervision. **Pingli Yang:** conceptualization, funding acquisition, resources, supervision.

## Funding

This study is supported by the 2024 Talent Development Fund (CZ001213).

## Ethics Statement

Approval was obtained from the Institutional Review Board of the First Affiliated Hospital of Shihezi University.

## Consent

Written informed consent was obtained from the participant enrolled in the study, including specific authorization for the publication of anonymized clinical data.

## Conflicts of Interest

The authors declare no conflicts of interest.

## Data Availability

The authors have nothing to report.
